# Sex, Drugs, and the Medial Amygdala: A Model of Enhanced Sexual Motivation in the Female Rat

**DOI:** 10.3389/fnbeh.2019.00203

**Published:** 2019-09-10

**Authors:** Sarah A. Rudzinskas, Katrina M. Williams, Jessica A. Mong, Mary K. Holder

**Affiliations:** ^1^Program in Neuroscience, University of Maryland, Baltimore, MD, United States; ^2^Department of Veterans Affairs, Geriatric Research Education and Clinical Center, Baltimore, MD, United States; ^3^Department of Pharmacology, University of Maryland, Baltimore, MD, United States; ^4^School of Psychology, Georgia Institute of Technology, Atlanta, GA, United States

**Keywords:** methamphetamine, dopamine, proceptive behavior, progesterone, sexual motivation, medial amygdala

## Abstract

Methamphetamine (METH) is a psychomotor stimulant that is reported to enhance sexual desire and behavior in both men and women, leading to increases in unplanned pregnancies, sexually-transmitted infections, and even comorbid psychiatric conditions. Here, we discuss our rodent model of increased sexually-motivated behaviors in which the co-administration of METH and the ovarian hormones, estradiol and progesterone, intensify the incentive properties of a sexual stimulus and increases measures of sexually-motivated behavior in the presence of an androgen-specific cue. We then present the neurobiological mechanisms by which this heightened motivational salience is mediated by the actions of METH and ovarian hormones, particularly progestins, in the posterodorsal medial nucleus of the amygdala (MePD), a key integration site for sexually-relevant sensory information with generalized arousal. We finally demonstrate the cellular and molecular mechanisms underlying this facilitation of sexual motivation by METH, including the upregulation, increased phosphorylation, and activation of progestin receptors (PRs) in the MePD by METH in the presence of ovarian hormones. Taken together, this work extends our understanding of the neurobiology of female sexual motivation.

## Introduction

Sexual behaviors are a complex, coordinated suite of actions that arise from the integration of psychological and physiological processes with external elements. One key component of sexual behaviors is that of sexual motivation, a hypothetical, internal willingness to engage in sexual behaviors (Holder and Mong, 2017). Although research into female sexual motivation is an active and growing field, relatively little is understood about the neurobiological origins of sexual motivation in women. Many of these mechanistic questions cannot be currently answered in women, so rat models are most frequently used to study sexual motivation and behavior (Pfaus et al., 2003; Blaustein, 2008).

In this review article, we discuss the modulators of female sexual motivation, using the concept of incentive motivation as a foundational working model. Next, we summarize what is known in regard to the neurobiology of female sexual motivation in rats. We then describe our methamphetamine (METH) model of increased sexually-motivated behaviors in female rats. We finally detail insights into the neurobiology and mechanisms of enhanced female sexual motivation gained using this model.

## Rodent Sexual Behaviors

The female rats show a wide range of specific sexual behaviors that are displayed in the presence of a male rat. Following anogenital investigations, the female will typically engage in approach and solicitation behaviors, which serve to initiate sexual contact with a male (McClintock and Adler, 1978; Erskine, 1989; Pfaus et al., 2003). The female approaches the male with a head-wise orientation then quickly runs away (Pfaus et al., 2003). This runaway takes the form of proceptive behaviors such as hopping and darting, with and without ear wiggling, in a traditional behavioral arena (Madlafousek and Hliňák, 1983). Hopping is distinct from general locomotion as it is a rapid, stiff-legged upward jump, followed by a bow-shaped return to the floor, and ends in a crouch. A hop covers the distance of approximately one extended body length (Madlafousek and Hliňák, 1977). Darting is a specialized form of a runaway from the male in which the female accelerates swiftly, using rapid low steps with the body held near the floor (Hemmingsen, 1933; Beach, 1942). The series of hopping-and-darting typically ends with a presentation behavior, or a pre-lordotic crouch (Madlafousek and Hliňák, 1977). This crouch serves to help support the male’s mounting behaviors. Upon a successful mount by the male, the female rat displays a behavioral reflex known as lordosis, in which the female arches her back, elevates her head and rump, and deflects her tail to one side (reviewed in Erskine, 1989). Proceptive behaviors typically precede the first lordosis during the period of sexual receptivity, and the numbers of proceptive events increase in the minute preceding lordosis (Chu and Ågmo, 2015). Indeed, females that display more proceptive behaviors are pursued more frequently by males (Chu and Ågmo, 2014). In addition, the female’s display of proceptive behaviors precedes nearly all male sexual behaviors (Bergheim et al., 2015).

In arenas that allow for separation between the male and female rat, such as a paced mating arena with escape chamber(s) or bilevel chambers, the female rat controls the tempo and occurrence of the sexual behaviors (McClintock and Adler, 1978; Erskine and Baum, 1982; Erskine, 1985; Pfaus et al., 2003). If female rats are given the opportunity to choose between two males, they display a consistent partner preference, as indicated by increased time with a preferred male and by returning to him more rapidly in a paced-mating environment even across multiple encounters (Lovell et al., 2007).

## Modulators of Sexual Motivation

### Central Motive State

**Figure 1:**
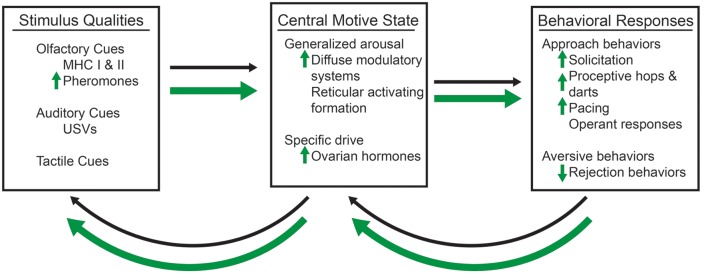
A conceptual model of sexual motivation, which are represented by thin, black arrows and enhancements by methamphetamine (METH), which are represented by thick, green arrows. The olfactory, such as the major histocompatibility complex I and II (MHC I and MHC II), which contribute to pheromones, auditory, such as ultrasonic vocalizations (USVs), and tactile, such as the flank stimulation, qualities of a sexual stimulus interact with the central motive state, which itself is modulated by the generalized arousal state and the activation of a specific drive (e.g., the presence of ovarian hormones and activation of their cognate receptors). METH requires the activation of dopaminergic and progestin receptors (PRs) to enhance the activation of the central motive state and female sexual motivation. The presence of a sexual stimulus and activation of the central motive state then influences the likelihood of sexual behavioral responses, which then have the ability to feedback and alter the central motive state itself. Changes to the central motive state then feedback to alter the salience of particular qualities of the sexual stimulus. METH increases the salience of androgen-specific cues of a sexual stimulus, which in term lead to increases in display of solicitations, proceptive hops and darts, and paced mating behaviors. There is also a decrease in the number of rejection behaviors.

There are two necessary components of any motivated behavior: (i) the incentive properties of an external stimulus; and (ii) a central motive state (Bindra, 1974; Ågmo, 1999). The external stimulus has incentive or aversive qualities that serve to influence the hedonic, or pleasurable values. Incentive stimuli create a tendency for an individual to approach the object; whereas, aversive stimuli create a tendency for avoidance behaviors (Bindra, 1974). The central motive state is the integration of the physiological processes, such as hormones, with the neural processes that direct the motivational behaviors (Bindra, 1974; Ågmo, 1999). It is the interplay between the incentive qualities of the stimulus and the central motive state that ultimately determine the likelihood of a particular behavioral response, whether it be approach or avoidance behaviors (Figure 1).

One major assumption of the central motive hypothesis is that of the hedonic value of the external stimulus. In order to apply this hypothesis to the study of female sexual motivation, it must, therefore, be established that females engage in sexual behavior to experience sexual pleasure (Pfaus et al., 2003; Ågmo, 2007). Sexually-motivated female rats lever press (Bermant, 1961; French et al., 1972), cross an electrified grid (Meyerson and Lindström, 1973), and nose-poke (Matthews et al., 1997; Cummings and Becker, 2012) to gain access to a sexually-active male. When female rats can pace sexual behavior, as in bilevel arenas or those with escape chambers, they show conditioned place preference in that they spend more time in the portion of an arena in which a sexual encounter occurred (Paredes and Alonso, 1997; Meerts and Clark, 2007). Subsequent studies indicate that female rats will only develop this conditioned place preference when copulation is at their preferred pacing interval (Jenkins and Becker, 2003b). Finally, female rats show evidence of orgasm-like behavior as indicated by contractions of the pelvic-floor muscles and short-term changes associated with reward state such as ultrasonic vocalizations (USVs; Pfaus et al., 2016). Taken together, these studies indicate that sexual behavior may, in itself, be rewarding to the female rat, at least under certain conditions.

The central motive state determines the external stimulus’s incentive value through modulation of the hedonic value of that stimulus (Berridge, 2004). It would then follow that changes to the central motive state could increase the attractivity to a sexually-relevant stimulus (Pfaus et al., 2003; Ågmo, 2007). That is, an increased activation of the central motive state would enhance the strength of behavioral responses toward sexually-salient cues. This may take the form of more olfactory investigations and displays of the solicitation, proceptive, and pacing behaviors in the presence of a male rat. In addition, it is possible that this enhanced activation of the central motive would also lead to the abolition of mate preferences, as the same sensory cues may increase in incentive qualities.

We can conceive of the central motive state as arising from two components: (i) a generalized state common to all forms of motivated behavior; and (ii) a specific drive that depends on physiological needs (Pfaff, 1999). The first component is generalized arousal which energizes all motivated behaviors (Pfaff et al., 2008). The specific neurobiological signals known to mediate the sexual motivation and behavior in the female rat are the ovarian hormones estradiol and progesterone (Cummings and Becker, 2012; Uphouse et al., 2015). Although these are not the only neurobiological factors that could alter the central motive state to lead differences in sexual motivated behaviors, we will focus discussion on the factors of generalized arousal and ovarian hormones in this review article.

### Generalized Arousal

Generalized arousal is a hypothetical construct that energizes all behavioral processes by promoting wake, alertness, and responses to and interaction with the environment (Pfaff et al., 2008). Generalized arousal has been demonstrated by: (i) a responsiveness to sensory stimuli across multiple modalities; (ii) motor activity; and (iii) emotional or affective reactivity (Pfaff et al., 2008). The ascending, diffuse neuromodulatory systems that form the reticular activating formation contribute to generalized arousal. Noradrenergic projections to the cerebral cortex modulate the sensory responsiveness, whereas the nigrostriatal dopaminergic projections mediate the motor activity directed towards salient stimuli (Pfaff et al., 2008). Mesolimbic dopaminergic projections, which comprise part of the natural reward circuit, facilitate the incentive salience, or the “wanting” of some stimulus (reviewed in Berridge, 2007, 2019). As such, certain types of sexual behaviors (e.g., female-paced sexual behavior) will result in a release of dopamine in the nucleus accumbens (Jenkins and Becker, 2003a). Further, the administration of agonists of noradrenergic or dopaminergic receptors will enhance, and antagonists will reduce, measures of female sexual behavior (Foreman and Moss, 1979; Fernández-Guasti et al., 1985a,b, 1987; Grierson et al., 1988; Petitti and Etgen, 1990; Chu and Etgen, 1999; Chu et al., 1999). Thus, both neurotransmitters appear to work in conjunction to modulate general arousal and prime a female towards sexual behavior.

### Ovarian Hormones

The period of sexual receptivity in rats is limited to a few hours prior to the onset of ovulation (Nequin et al., 1979; Freeman, 1994). Several classic studies have demonstrated the role of both estradiol and progesterone in triggering both proceptive and receptive sexual behaviors in the rat (Beach, 1976). High levels of estradiol are sufficient and activation of the estrogen receptors (ERs) is necessary to induce lordosis behaviors; however, the intensities of lordosis, based on the degree of spinal curvature, is highly variable with frequent displays of rejection behaviors (Boling and Blandau, 1939; Beach et al., 1942; Whalen, 1974; Spiteri et al., 2010). Progesterone increases the efficacy of estradiol in the induction of lordosis. In addition, progesterone and the activation of the PRs is necessary for the occurrence of the solicitation, proceptive, and paced mating behaviors (Boling and Blandau, 1939; Beach et al., 1942; Beach, 1976; Whalen, 1974; Fadem et al., 1979; Tennent et al., 1980; Edwards and Pfeifle, 1983; Olster and Blaustein, 1988; Blaustein, 2008). These hormones strongly affect the responses to olfactory and tactile stimuli, with modest effects on generalized arousal (Chu et al., 2015), providing evidence that the ovarian hormones contribute to the central motive state to modulate the incentive qualities of the male rat.

## Neurobiology of Sexual Motivation

The historical focus of the neurobiology of female sexual behavior has been focused on the neurocircuit that controls lordosis. As lordosis is a behavioral reflex, the neural mechanisms of it are more readily elucidated than the neural mechanisms of sexual motivations. The lordosis circuit has been exquisitely detailed using multilateral approaches including electric stimulation and lesions of each of the nuclei in the circuit (Mathews and Edwards, 1977; Davis et al., 1979; Pfaff and Sakuma, 1979; Sakuma and Pfaff, 1979; Brink and Pfaff, 1980; Schwartz-Giblin and Pfaff, 1980; Femano et al., 1984a,b), patterns of neuronal activation (Flanagan et al., 1993; Tetel et al., 1993; Flanagan-Cato and McEwen, 1995; Polston and Erskine, 1995; Pfaus et al., 1996; Pfaus and Heeb, 1997), and viral tract tracing studies to map the anatomical connections (Daniels et al., 1999). Of primary importance for lordosis is the ventrolateral portion of the ventromedial nucleus of hypothalamus (VMN; reviewed in Pfaff et al., 1994). The ovarian hormones serve to activate the neurons of the VMN, which then overcomes the tonic inhibition on lordosis (Powers and Valenstein, 1972; Moss et al., 1974; Pfaff and Sakuma, 1979; Kow et al., 1985; Fahrbach et al., 1989).

The mechanisms and the neural circuitry controlling female sexual motivation have not been as well elucidated. Furthermore, if motivated behavior arises from both the incentive properties of a sensory stimulus and mediators of the central motive state, it is likely that the neural circuitry that processes these sensory cues also contribute to sexual motivation. The work of ourselves and others indicates that sexual motivation arises from an interplay of activation of the natural reward circuity and the processing of olfactory cues in the limbic/hypothalamic social behavior circuitries.

The posterodorsal nucleus of the medial amygdala (MePD) is a good candidate region for the regulation of sexual motivation and the modulation of the output sexual behavior (Mascó and Carrer, 1980, 1984; Erskine, 1989; Kondo and Sakuma, 2005; Afonso et al., 2009). Changes to generalized arousal would influence the activation of the MePD as it receives both noradrenergic and dopaminergic input (Gray, 1999; Pitkänen, 2000). The MePD contains both ERs and PRs (Pfaff and Keiner, 1973; Simerly et al., 1990), making it sensitive to the specific drivers of sexual motivation. The MePD also receives chemosensory signals of pheromones from the accessory olfactory bulb (Keller et al., 2009), so it would be activated by sexually relevant olfactory cues. The projections of the MePD target and can activate several key output nuclei involved in social and sexual behaviors including the VMN (Kevetter and Winans, 1981; Simerly, 2002; Keller et al., 2009). Finally, lesions of the MePD lead to fewer lordosis responses (Mascó and Carrer, 1984), proceptive behaviors (Mascó and Carrer, 1980; Afonso et al., 2009), and a reduction in conditioned place preference (García-Horsman et al., 2008) and sensitivity to sexual stimulation (Guarraci, 2010).

## Methamphetamine Increased Measures of Sexual Motivation Towards an Incentive Stimulus

To better explore the neurobiology of sexual motivation in females, we created a model of enhanced motivation by administering METH. METH is a drug of abuse that intensifies sexual drives, desires, and sexual activities in women (Rawson et al., 2002; Semple et al., 2004a). In addition, METH use is also associated with a more pleasurable sexual experience (Lorvick et al., 2012). These anecdotal and clinical self-reports are supported by the increased rates of sexually-transmitted infections and of unplanned pregnancies (Semple et al., 2004b; Mansergh et al., 2006). As users of METH tend to administer it several times over the course of a few days (Haile et al., 2009), we administer METH (5 mg/kg/day) once a day for 3 days to ovariectomized female rats at the same time as the ovarian hormones estradiol benzoate and progesterone (Holder et al., 2010). The optimal time to test for female sexual behavior is 4–6 h following the administration of progesterone (Nequin et al., 1979; Freeman, 1994). Importantly, neither stereotyped behavior nor hyper-locomotor behavior are present 4–6 h after METH administration, suggesting that any increase in sexual behavior due to METH reflects heightened sexual motivation, not motor responses (Holder et al., 2010).

**Figure 2:**
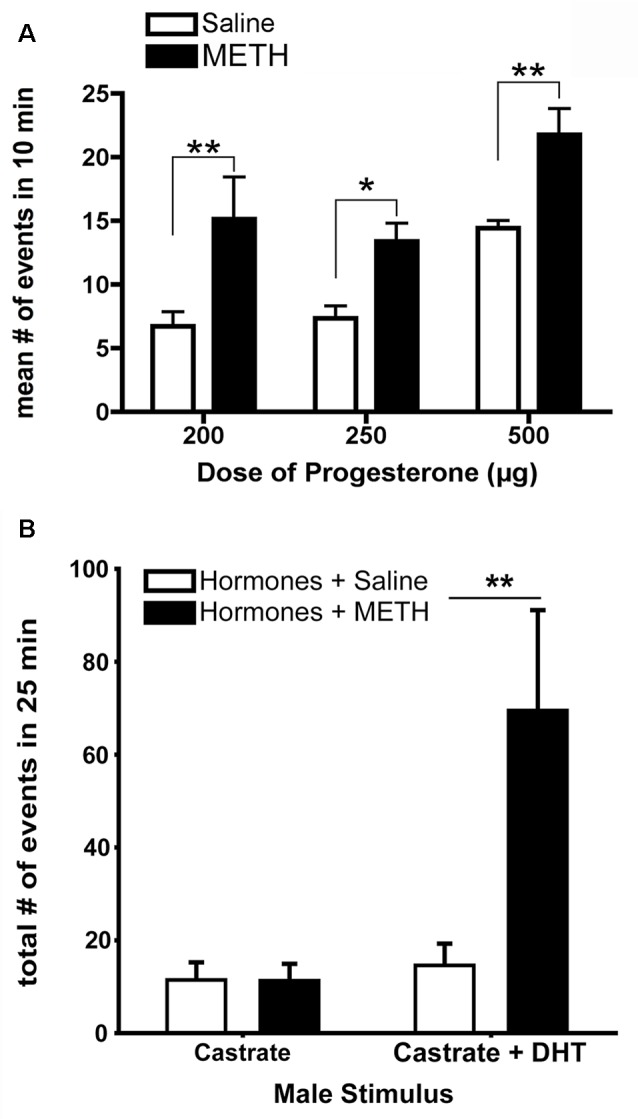
The enhancement of sexually-motivated behaviors by METH. **(A)** METH treatment doubles the number of proceptive events displayed in 10 min regardless of progesterone (P) dose, compared to the respective saline-treated females. **(B)** Replacement of androgen-specific cues in castrated males induces more markedly increased proceptive behaviors in hormonally-primed female rats treated with METH. Data represents means ± standard error of the mean (SEM); *p < 0.05, **p < 0.01. Reprinted with permission from Elsevier, Inc., Amsterdam, Netherlands.

The acute administration of METH enhances measures of sexual motivation in hormonally-primed female rats (Holder and Mong, 2010; Holder et al., 2010; Winland et al., 2011). METH treatment increases the lordosis response in addition to doubling the frequency of proceptive behavior of hops, darts, and ear-wiggles (Figure 2A; Holder et al., 2010). When tested in a paced-mating arena, female rats treated with METH are less likely to leave the male rat following sexual stimulation, and if they leave, they return to him more rapidly compared to saline-treated, hormonally-primed females (Holder et al., 2010; Winland et al., 2011). In addition, these METH-treated female rats displayed more solicitation and proceptive behaviors, especially during the post-ejaculatory interval (Holder and Mong, 2010). The possibility remains that METH may alter the timing and displays of sexual behavior instead of sexual motivation *per se*; however, there is growing evidence that motivation and timing of behaviors are not independent processes such that changes to the hedonic value lead to alterations in interval duration, indicating that the changes in timings of a behavior are produced by changes in motivational state (reviewed in Galtress et al., 2012). While the decreased latency to return to the male is suggestive of an increased tempo for sexual behavior, the timing aspects should be further explored using more direct measures of sexual motivation in female rats (e.g., operant responding).

METH may also alter the preferences of specific sexual partners based upon relevant sensory cues. For example, METH-treated, hormonally-primed female rats make more approaches and spend more time with a potential sexual partner (e.g., a male or a castrated male treated with dihydrotestosterone) compared to a non-sexual partner (e.g., a female or a castrated male; Winland et al., 2011; Rudzinskas and Mong, 2016). Dihydrotestosterone provides the necessary androgen-mediated cues, such as pheromones (Orsulak and Gawienowski, 1972; Drewett and Spiteri, 1979), sufficient to elicit solicitation, hops, and darts, with METH treatment increasing the number of proceptive behaviors (Figure 2B; Rudzinskas and Mong, 2016). These pheromonal cues are olfactory in nature, and while there are no differences in anogenital investigations induced by METH, there are significantly fewer sniffing behaviors. Consistent with an increase in generalized arousal as part of the central motive state, this work suggests that METH may enhance the detection of olfactory cues. Future work is necessary to explore the potential effects of METH on olfaction. Taken together, these data suggest that METH does not alter the ability of females to discriminate between stimuli, but rather enhances central motive state arousal to increase sexual motivation in a context-specific manner by potentiating the behavioral responses towards an incentive stimulus.

## A Locus for Enhanced Sexual Motivation

**Figure 3:**
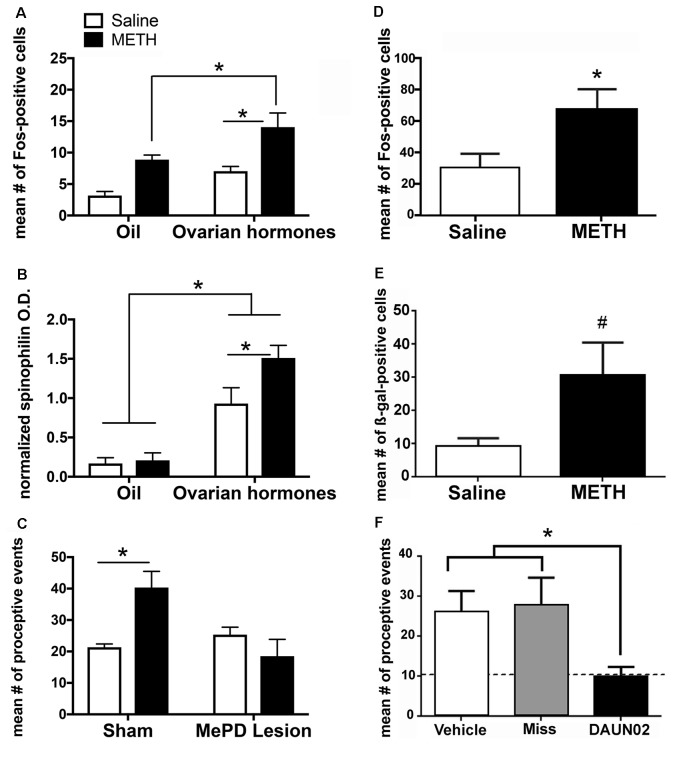
The posterodorsal medial amygdala (MePD) is a locus for the enhancement of sexual motivation by METH. **(A)** The combination of METH and the ovarian hormones estradiol and progesterone increases Fos-immunoreactivity in the MePD, compared to either METH-oil controls and saline-hormone controls. **(B)** METH treatment significantly increases spinophilin protein levels, compared to saline-hormone controls. **(C)** There was a significant interaction of METH and the MePD lesion, such that the lesion of the MePD blocks the METH-induced increases in proceptive behaviors. **(D)** METH increases Fos-immunoreactivity in the presence of ovarian hormones in cFos-lacZ transgenic rats. **(E)** There is a strong trend towards an increase in β-galactocidase (β-gal)-immunoreactivity in the presence of both ovarian hormones and METH in cFos-lacZ transgenic rats. **(F)** DAUN02 inactivation of ovarian hormone- and METH-responsive cells in the MePD prevents the METH-induced increase in proceptive behavior, compared to vehicle-controls and animals in which DAUN02 is infused into areas other than the MePD (Miss). The dashed line represents the baseline levels of proceptive behavior induced by ovarian hormones. Data represents means ± SEM; *p < 0.05, ^#^*p* = 0.05. Reprinted with permission from Elsevier, Inc., Amsterdam, Netherlands; **(A–C)** and under the use of the Creative Commons license **(D–F)**.

The combination of METH and ovarian hormones enhances the measures of sexual motivation; therefore, we hypothesized that METH would converge with ovarian hormone actions to increase neuronal activity and induce neuroplasticity of the neurocircuitry that underlies sexual motivation and behavior. There is an additive effect of METH and ovarian hormones on the expression of cFos, an immediate early gene that is used as a marker of neuronal activation, in both the MePD and VMN (Figure 3A; Holder et al., 2010; Williams and Mong, 2017). The MePD projects to and can activate the VMN (Kevetter and Winans, 1981; Simerly, 2002; Keller et al., 2009). Therefore, it is likely that the increase in cFos in the VMN follows the increase in neuronal activation of the MePD. In further support, spinophilin, a cytoskeleton-associated protein found in dendrite spines, has a 60% increase in the MePD, but not the VMN, following the administration of METH and ovarian hormones (Figure 3B; Holder and Mong, 2010). This increase in spinophilin suggests that METH and ovarian hormones synergize to increase the density of dendritic spines and, thus, synaptic connectivity in the MePD. Taken together, the increase in neuronal activation and spinophilin in the MePD suggest that the METH-induced enhancement of female sexual motivation and behavior arise from converging actions of the ovarian hormone in the MePD.

It has been previously reported that lesions of the MePD do not abolish the expression of female sexual behavior, but rather, reduces the expression of sexually-motivated behaviors (Mascó and Carrer, 1980, 1984; Afonso et al., 2009). Lesions of the MePD also prevent the METH-induced increase in proceptive behaviors (Figure 3C; Holder et al., 2015). The Daun02 inactivation techniques allow for a more precise investigation of the cells activated in the MePD and the interactions of METH and ovarian hormone signaling on sexually motivated behaviors. Briefly, neuronal activation induces both cFos and β-galactosidase expression in Sprague–Dawley cFos-lacZ trans-genetic rats (Koya et al., 2016). The β-galactosidase both serves as another method of visualizing activated neurons, but it also converts the prodrug Daun02 into daunorubincin, which then triggers apoptosis of the activated cell populations (Santone et al., 1986; Farquhar et al., 2002; Pfarr et al., 2015). Therefore, this Daun02 inactivation technique produces selective lesions of the cells that are activated by METH and/or ovarian hormones. As with the cFos expression, the combination of METH and ovarian hormones produces an increase of β-galactosidase over that of ovarian hormones in a discrete population of cells within the MePD (Figures 3D,E; Williams and Mong, 2017). In addition, successful Daun02 lesions of this neuronal ensemble reduce the proceptive behaviors to baseline levels, further supporting the notion that the MePD utilizes signals from METH on hormonally responsive neurons to augment the behavioral response (Figure 3F; Williams and Mong, 2017). Furthermore, the Daun02 inactivation does not alter receptive/reflexive sexual behaviors, supporting the MePD’s role as an integration center specifically for sexual motivation. Taken together, these results indicate a synergy of intracellular signaling cascades induced by METH and ovarian hormones within MePD cells. This will be further explored in subsequent sections.

One way in which this synergy of intracellular signaling cascades could result in an increase in sexual motivation is *via* changes in epigenetic modifications, which can then lead to marked changes in gene expression. These epigenetic changes could occur on the DNA directly, leading to localized regulation of gene transcription, or by modification of the histones, an integral part of the chromatin around which the DNA spools, which lead to more global alteration of gene transcription (reviewed in Robison and Nestler, 2011). DNA methylation, in which methyl groups are added to DNA molecules by DNA methyltransferase (DNMT), results in repression of gene transcription. Both METH and ovarian hormones reduce the enzymatic activity of DNMT in the MePD (Rudzinskas and Mong, 2018). Acetylation of the histones enables gene transcription by allowing chromatin expansion, and histone deacetylases (HDAC) are enzymes that remove the acetyl groups, leading to more tightly coiled DNA and a reduction of gene transcription. The combination of both METH and ovarian hormones reduces the enzymatic activity of HDAC in the MePD (Rudzinskas and Mong, 2018). Reduced activity of both HDAC and DNMT should allow for enhanced gene transcription in cells of the MePD. Moreover, these data further support the notion that the MePD is a locus for this enhanced sexual motivation, as no significant changes in HDAC or DNMT activity occur in the VMN. In addition, these changes in enzymatic activity are not the result of changes in the total protein levels of the enzymes (Rudzinskas and Mong, 2018). Taken together, these data suggest that dynamic epigenetic changes may play some role in the genetic mechanisms which underlie METH-enhanced proceptivity. As such, these changes should be investigated further on a gene-by-gene basis, particularly in relationship to the genes explored in the next section of this review article.

## Mechanisms of Enhanced Sexual Motivation

### Dopamine Receptors

While cells within the MePD mediate the METH-facilitated increase in proceptive behavior, it remains unclear how METH and ovarian steroids specifically activate this cellular population to increase neural activation. One likely source of neural activation is dopamine, as one of the primary responses following METH administration is the release of a bolus of dopamine into the synapse (Sulzer et al., 2005; Fleckenstein et al., 2007). The MePD receives both direct and indirect inputs from the ventral tegmental area, a major source of dopaminergic synthesis in the mesolimbic, natural reward pathway (reviewed in Ikemoto, 2007). Thus, it is likely that dopamine receptor (DR) activation in the MePD mediates the enhanced sexually motivated behaviors by METH.

**Figure 4:**
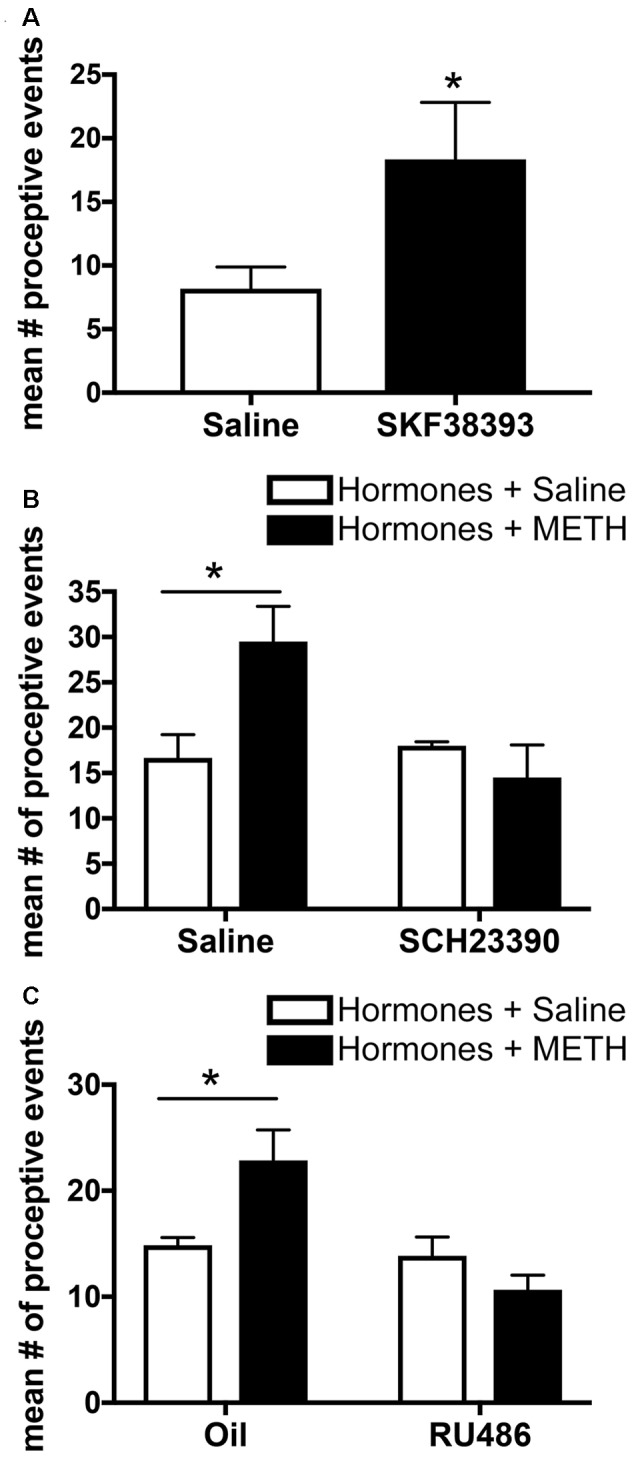
Effects of microinfusions into the posterodorsal medial amygdala (MePD) on sexually-motivated behaviors. **(A)** Infusion of the dopamine type-1 receptor (D1R) agonist SKF38393 increases the number of proceptive events displayed in 10 min. **(B)** Infusion of the D1R antagonist SCH23390 prevents the METH-induced increase in proceptive behaviors. **(C)** Infusion of the progestin receptor (PR) antagonist RU486 prevents the increase in proceptive behavior induced by METH treatment. Data represents means ± SEM; *p < 0.05. Reprinted with permission from Elsevier, Inc., Amsterdam, Netherlands.

Activation of the excitatory D1-type DRs (D1Rs), which comprise both D_1_R and D_5_R, in the MePD in the absence of METH increases the number of proceptive events above levels induced by ovarian hormones alone (Figure 4A). In addition, administration of an antagonist to these D1Rs in the MePD prevents the METH-induced increase in proceptive behaviors (Figure 4B; Holder et al., 2015). In contrast, administration of agonists or antagonists to the D2-type DRs, which comprise D_2_R, D_3_R and D_4_R, in the MePD has no effect on the number of proceptive behaviors displayed. Interestingly, the overall quantity of D1Rs in the MePD remained unchanged between treatment groups (Rudzinskas, 2017). These experiments suggest that METH, through a release of dopamine, may work through the activation of a stable population of D1Rs in the MePD to directly modify the expression of genes underlying female sexual motivation.

The DR agonists and antagonists were administered once a day, for 3 days; therefore, it is probable that the genes affected would be both those necessary for the immediate display of sexual motivation and behavior and those involved in more long-lasting changes to the sexual motivation circuit. One such gene whose expression could be modified *via* changes in D1R activation is the PR (Olesen et al., 2005, 2007). Indeed, METH alone, in the absence of estradiol, increases PRs in the MePD (Holder et al., 2015). However, in a follow-up study, a D1R antagonist infused into the MePD at the same time of METH administration failed to change PR levels in the MePD (Williams et al., 2018). It may be microinfusions of the antagonist at a longer time course prior to METH administration may have prevented the METH-induced increase in PRs.

### Progesterone Receptors

Progesterone and activation of the PRs are necessary for the display of proceptive behavior, and there is functional specificity of the two isoforms of the nuclear receptor. PR_A_ activation contributes primarily to the display of lordosis, whereas PR_B_ activation seems to contribute primarily to proceptivity (Mani et al., 2006). PR activation, primarily through PR_B_, in the MePD may facilitate increases in proceptive and other sexually-motivated behavior. While the contributions of the PR isoforms in the MePD to enhancement of sexual motivation by METH has not been determined, it has been demonstrated that the microinfusion of RU486, a PR antagonist, into the MePD decreases the METH-facilitated proceptive behaviors (Figure 4C; Holder et al., 2015). Finally, recent work demonstrates that increasing PR protein expression with a lentiviral overexpression vector injected into the MePD in the absence of METH increases proceptive behaviors and lordosis intensity, with no other noted effects on social, exploratory, or rejection behaviors (Williams et al., 2018). Taken together, it is clear that PRs in the MePD have functional relevance toward the induction of female sexual motivation.

### Intracellular Mechanisms

In addition to activated D1R increasing the number of PRs in the absence of estradiol, D1R activation can also activate PRs in the absence of progesterone (Auger, 2001); therefore, METH-facilitated activation of D1R could work *via* other, intracellular mechanisms in conjunction with the increased PRs to enhance sexual motivation. The ligand-bound PR is necessary to modulate proceptive behaviors; however, METH can facilitate these proceptive behaviors even in the presence of subthreshold doses of progesterone (Figure 2A; Holder et al., 2010). Taken together, this evidence suggests that METH administration enhances PR sensitivity to ligand in the MePD (Weigel et al., 1995).

The changes to PR sensitivity and/or functionality may arise from post-translational modifications, which include phosphorylation, acetylation, sumoylation, and ubiquitination (Hagan et al., 2012). Of these, the phosphorylation is thought to be the primary regulator of PR actions, such that phosphorylation of specific sites on the PR enhances transcriptional activity (Denner et al., 1990; Bai et al., 1997; Weigel and Moore, 2007). In fact, activation of D1Rs leads to a sequence of kinase phosphorylation events, which could then modulate the activational state of the PRs (Auger, 2001). The PR is highly promiscuous, as it is able to dock onto activated mitogen-activated protein kinases, such as the extracellular signal-regulated kinases (ERK1/2), Src kinases, and the ERs (Lu and Xu, 2006; Dressing et al., 2009). ERK1/2 has been reported to directly phosphorylate the progesterone receptor, while both ERK1/2 and Src kinase have been reported to act in a complex with both ERs and PRs (Migliaccio et al., 1998; Boonyaratanakornkit et al., 2001). The activation of these kinase cascades leads to enhancements of both receptive and proceptive behaviors (González-Flores et al., 2009, 2010; Lima-Hernández et al., 2012).

**Figure 5:**
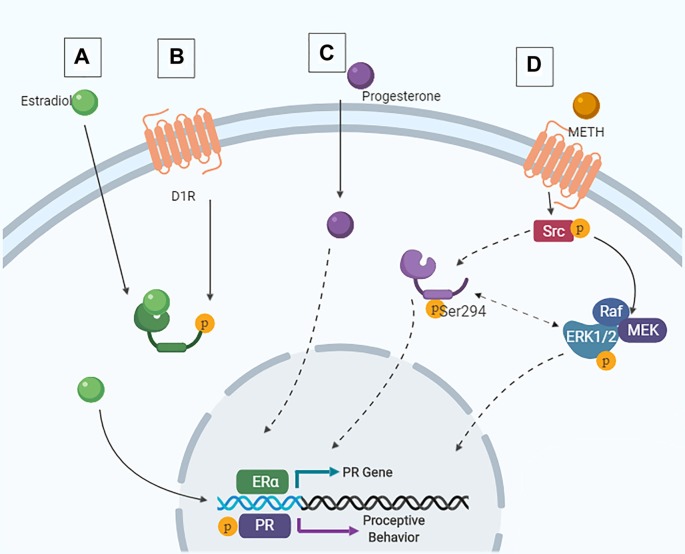
Intracellular signaling cascades in the MePD that contribute to female sexual motivation. **(A)** Estradiol enters the cell and binds to estrogen receptor (ER), leading to ligand-dependent gene transcription. **(B)** Following METH-induced dopamine release, signaling *via* the D1R also leads to ER translocation to the nucleus and ligand-independent transcription of the PR. These are the priming stages. **(C)** Similarly, progesterone can enter the cell and lead to ligand-dependent signaling. **(D)** Following activation of the D1R, Src interacts with the PR, leading to phosphorylation of Src and PR at Ser294. This results in downstream ERK1/2 phosphorylation and increases proceptive behavior.

As the activation of both D1Rs and PRs in the MePD are necessary for the METH-induced enhancement of proceptive behaviors, it is likely that the behaviorally-relevant neurons contain both D1Rs and PRs and that the METH-induced enhancement of sexual motivation arises due to the activity of kinases. In support, METH administration leads to phosphorylation of the ubiquitous kinases ERK1/2 and cSrc in hormonally intact or primed rats (Hebert and O’Callaghan, 2000; Choe et al., 2002; Zhang et al., 2004; Pascoli et al., 2005; Williams et al., 2018). The cytosolic-dependent kinase pathways that could be induced by D1R activation converge with the hormone-dependent kinase pathways at two serine sites in the PRs, serine 294 and 345, suggesting a molecular mechanism through which METH may modulate PR activity (Figure 5).

The combined actions of METH and the ovarian hormones increased the phosphorylation of PR serine 294, but not serine 345, in the MePD. Moreover, the administration of a D1R antagonist prevented this increase in phosphorylation of the PR_B_ at serine 294 (Williams et al., 2018), further supporting the role of the PR_B_ isoform in the mediation of proceptive behaviors. The activity of the Src kinase to phosphorylate serine 294 of the PR is required for the enhancements of sexual motivation of METH; however, blocking the activation of the ERK1/2 also prevents the METH-induced increases of proceptive behaviors without affecting the serine 294 phosphorylation. Taken together, these data provide evidence of a direct molecular interaction of D1R and PR actions such that the intracellular signaling cascades initiated by D1R activation phosphorylate a site on the PRs in order to modulate the activational states of the PRs. Further studies are necessary to elucidate the role of serine 294 in the MePD and in the relative contributions of the different kinase activation pathways in the MePD on the enhancement of sexual motivation in the female rat. The utilization of modern tools such as CRISPR/Cas-9 may provide further insights into the specificity of this signaling cascade as it relates to female sexual motivation. Ultimately, though, the activation of phosphorylation kinases and enhanced activation of PR would lead to an increase in the transcription of PR-dependent genes. The gene targets of the activated PRs in the MePD that enhance female sexual motivation and influence the central motive state have yet to be determined.

## Conclusion

One of the key components for sexual behavior is that of sexual motivation. We have presented one model system in which we can further study the motivational aspects of sexual behavior. The data presented in this review article indicates that sexual motivation arises from interactions of neurotransmitters and steroid hormones to change the central motive state. In addition, these interactions can be influenced by pharmacological agents, such as METH, to further increase the central motive state and drive the response to sexually-relevant stimuli. With the advent of technologies that enable us to examine and determine the nature of these interactions on epigenetic and molecular levels, we approach answers to such fundamental questions as the origins of sexual motivation. The use of the METH-model of enhanced sexually-motivated behaviors has already revealed complexities to an admittedly intricate and multifaceted system; however, this model also presents new avenues for research that may ultimately reveal the origins of sexual desire.

## Author Contributions

SR, KW, JM, and MH designed the studies referenced. SR, KW, and MH performed the experiments and wrote sections of the manuscript. MH wrote the outline of the manuscript. All authors contributed to manuscript revision, read and approved the submitted version.

## Conflict of Interest Statement

The authors declare that the research was conducted in the absence of any commercial or financial relationships that could be construed as a potential conflict of interest.
